# Towards interactive explanation-based nutrition virtual coaching systems

**DOI:** 10.1007/s10458-023-09634-5

**Published:** 2024-01-20

**Authors:** Berk Buzcu, Melissa Tessa, Igor Tchappi, Amro Najjar, Joris Hulstijn, Davide Calvaresi, Reyhan Aydoğan

**Affiliations:** 1 Computer Science, Özyeğin University, Istanbul, Turkey; 2 Computer Science, High National School of Computer Science ESI ex-INI, Algiers, Algeria; 3 Luxembourg Institute of Science and Technology, Esch-sur-Alzette, Luxembourg; 4 University of Luxembourg, Esch-sur-Alzette, Luxembourg; 5 University of Applied Sciences and Arts Western Switzerland (HES-SO Valais-Wallis), Sierre, Switzerland; 6 Interactive Intelligence, Delft University of Technology, Delft, The Netherlands; 7 University of Alcala, Alcala de Henares, Spain

**Keywords:** Explainable AI, Recommender systems, Interactive, Nutrition virtual coach

## Abstract

The awareness about healthy lifestyles is increasing, opening to personalized intelligent health coaching applications. A demand for more than mere suggestions and mechanistic interactions has driven attention to nutrition virtual coaching systems (NVC) as a bridge between human–machine interaction and recommender, informative, persuasive, and argumentation systems. NVC can rely on data-driven opaque mechanisms. Therefore, it is crucial to enable NVC to explain their doing (i.e., engaging the user in discussions (via arguments) about dietary solutions/alternatives). By doing so, transparency, user acceptance, and engagement are expected to be boosted. This study focuses on NVC agents generating personalized food recommendations based on user-specific factors such as allergies, eating habits, lifestyles, and ingredient preferences. In particular, we propose a user-agent negotiation process entailing run-time feedback mechanisms to react to both recommendations and related explanations. Lastly, the study presents the findings obtained by the experiments conducted with multi-background participants to evaluate the acceptability and effectiveness of the proposed system. The results indicate that most participants value the opportunity to provide feedback and receive explanations for recommendations. Additionally, the users are fond of receiving information tailored to their needs. Furthermore, our interactive recommendation system performed better than the corresponding traditional recommendation system in terms of effectiveness regarding the number of agreements and rounds.

## Introduction

Approximately 63% of all deaths worldwide are attributed to non-communicable diseases such as cardiovascular diseases, chronic respiratory diseases, and diabetes.^1^ The World Health Organization emphasizes that these diseases can be prevented by addressing common risk factors, such as unhealthy nutrition habits and diets. However, personal preferences, cultural and religious constraints, and taste heavily affect individuals’ habits. Tasty—yet unhealthy components—are increasingly hidden in a wide range of processed food items. Therefore, society needs guidance on making suitable and sustainable dietary choices [8, 12, 44]. To counter the unhealthy trend, food recommender systems—assisting individuals in recipe selection have gained popularity [8, 44]. The need for these systems can be attributed to increased globalization, leading to greater availability and variety of food, as well as the prevalence of ultra-processed food, contributing to metabolic and overweight issues [18]. Although numerous recipes are freely accessible (i.e., via many online collectors), determining the “best” recipe for a *specific* individual in a *given* situation can be remarkably complex. Indeed, it involves managing a wide range of possibilities while considering bounding variables such as allergens, nutritional values, personal requirements, calorie intake, historical data, and momentary preferences. Consequently, there is a need for a personalized support system. Nutrition virtual coaches (NVCs) are systems that aim to recommend recipes that align with users’ specific needs and preferences while considering their health and long-term needs [43].

NVCs cater to various goals, including muscle gain, weight loss, and management of nutrition-related diseases such as obesity.^2^ The underlying objective is to provide users with constructive “educational” support, gradually reducing their reliance on NVCs. Existing solutions, both from research and industry, have attempted to address these goals. However, they often lack transparency and clarity, leading to a lack of trust and effectiveness [8]. To enhance transparency and, henceforth, effectiveness, Explainable AI (XAI) techniques have been employed in various domains, such as transportation, fleet management, and neurosciences [14, 33]. Moreover, some studies have proposed semantic models [34] and incorporated negotiation techniques to guide users towards desired quality of life goals [28].. While these efforts have contributed to the field of recommender systems, to the best of our knowledge, there is currently no existing system that fully qualifies as an “explainable” Nutrition Virtual Coach (NVC) which is effectively an agent that provide recommendations, explain them to the user, and engage in interactive discussions to foster desired behavioral changes. Engaging the user in interactive (back-and-forth) communication is crucial as it allows the user to dive into the concept and build a more solid and backed-up knowledge/awareness that undoubtedly boosts information retention. Such mechanisms can assume a rather simplistic—yet effective—form of feedback [27]. Building on that, verifying/fixing misunderstandings and elaborating on follow-up questions becomes more feasible (from a designer/developer perspective) and easy to handle (from a user perspective).

This work builds upon the protocol described in [6], and it extends it by introducing a more sophisticated/dynamic explanation generation strategy consisting of decision trees in the form of Item and User based trees to generate explanations retroactively to recommendation selection. Moreover, we have improved the user interface, leveraging the feedback coming from the user study conducted in [6]. Finally, we have extended the comparative evaluation of the proposed system using a simple health score calculation, with a multi-criteria additive utility function for recipe selection and an Web Ontology Language (OWL) based ontology database to classify users and recipe ingredients.

Our main assumption is that people can have different preferences (i.e., taste over healthiness or vice-versa). However, recommender systems, in prioritizing recommendations aligned with predefined goals, may sometimes overlook specific user preferences, leading to “conflicts” between user desires and system objectives. For instance, a user seeking tasty yet conversely unhealthy food may clash with a system focused on promoting a healthy lifestyle. The system developers in that case must delicately balance meeting the system goals while delivering a personalized experience. Therefore, to address these conflicts, we model the resolution as a negotiation in a dialogical setting where the system concedes by making recommendations more fitting to the user profile than its own goals (healthiness). We classified the participants according to their priorities (obtained via a pre-experiment survey). Moreover, we assessed the protocol with individuals characterized by various backgrounds in online experimental settings consisting of a pre-experiment survey, two sessions (static vs. interactive), and a concluding post-experiment survey to question the participants about their experience with the different settings.

The rest of this paper is organized as follows. Section 2 presents the related work. Section 3 presents the explainable argumentation negotiation module for NVC. Section 4 evaluates and discusses the obtained results. Finally, Sect. 5 concludes the paper and outlines future works.

## Related work

This section briefly overviews the literature on food recommender systems, focusing on conventional systems and their evolution to embrace explainable and interactive recommendations.

### Conventional food recommendation

In 1986, Hammond et al. [21] developed one of the earliest food recommender systems. It is named CHEF and leverages case-based planning to replace or improve food items within recipes. It requires a substantial initial knowledge base, extensive pre-processing, and the creation of (backup) plans for each recipe. More recently, in 2010, Freyne and Berkovsky [16] implemented recommender algorithms, such as collaborative filtering (CF) and content-based (CB) approaches, to recommend recipes. The study concluded that incorporating ingredient weights within CF and CB improved prediction accuracy. In turn, Ge et al. [17] introduced the concept of personalization in food recommendations, prioritizing health over taste. Chi et al. [11] focused on recommending food for individuals with chronic conditions (i.e., kidney diseases) using an Ontology Web Language (OWL) ontology integrating health-relevant aspects. Chen et al. [10] proposed a generalized framework for healthy recommendations, explicitly targeting the modification of unhealthy recipes. The authors introduced a deep learning-based method called IP-embedding to match recipes with desired ingredients, creating a pseudo recipe that meets the requirements and then matching it with healthy ingredients and real recipes using the mean squared error (MSE) metric. Similarly, Teng et al. [39] developed a point-wise comparison metric to understand how to transform recipes into more healthier ones, using ingredient substitutions for healthier alternatives. Elsweiler et al. [1] addressed ingredient and food substitution, metricizing nutritional values to encourage users to prefer healthier options. Overall, food recommendation approaches often rely on factors such as recipe content (e.g., ingredients) [13, 15, 40], user behavior history (e.g., eating history) [32, 46], and dietary preferences [32, 45].

### Towards explainable recommendation systems

Conventional food recommendation approaches are mostly “one-shot”, offering the user minimal (if any) possibilities to interact. However, with the advent of explainable technologies, that aim for predictors and classifiers that show transparency, understandability, and inspectability in order to boost trust [4], recommender systems are expected to provide explanations for their recommendations [13, 15, 20, 46], allowing users to justify, control, and discover new aspects of the suggested outcomes [32, 45, 47]. Along this line, Padhiar et al. [34] proposed a food recommender system that generates explanations based on a knowledge-based ontology. However, the explanatory system only attempts to explain a given recommendation via different methods, with no dialogue option: no way for the user to reply or interact. Samih et al. [36] further explored this concept by developing a knowledge-based explainable recommender system that makes use of a probabilistic soft-logic framework to generate explanations. Lawo et al. [28] aimed to enhance the interaction between users and virtual assistants by incorporating a cluster of consumers with ethical and social priorities into the recommendation process and considering their feedback and preferences.

Finally, recommendation systems have been employed in the nutrition domain for some time, with objectives ranging from promoting health, sustainability, and finding combinations of ingredients that taste well. Recent studies have emphasized the importance of incorporating explanations into recommendations to enhance transparency, trust, and acceptability. Although explanations in food recommender systems are still not fully widespread, some approaches (or combinations of them) are gaining attention. In the following section, we survey existing explanation mechanisms, which could be adopted by food recommendation systems.

### Post-hoc explanation generation mechanisms

In recent years, there has been ample research within the Machine Learning literature, focused on developing techniques for post-hoc explanation generation in various domains. These techniques are designed to explain the predictions made by complex black-box models. They operate “post-hoc,” meaning they generate explanations after the main model has made its predictions, without requiring modifications to the underlying architecture or training process. The goal is to improve transparency and interpretability by providing human-understandable justifications for the model’s decisions. Post-Hoc Explanation Generation models leverage techniques such as feature importance analysis [35], rule-based reasoning [50], gradient-based attribution [3], or surrogate models [51] to generate meaningful explanations that can shed light on the factors influencing the model’s predictions. These explanations help stakeholders gain insight into how the given model arrives at its decisions, builds trust, and facilitates error analysis, making them valuable tools for practical applications and model understanding [9].

We can distinguish various strategies to generate explanations. Note that these classes are not mutually exclusive but are often overlapping. So a user-centred explanation can also be content-based. The most suitable forms of explanations presented in the literature to be generated for food recommendations, can be classified as follows:

- *User-centered explanation:* The generated explanations are meant to assist users in achieving their goals. Sovrana and Vitali  [38] emphasize that users are satisfied with the explanations if they are guided in answering the questions about the process of fulfilling their goals. An explanation such as “*we recommend you the following food recipe to lose weight since it has low fat and rich fiber*” could be considered an instance of this explanation type. It implicitly answers what is necessary to lose weight, which aligns with the hypothetical user’s goals.
- *Knowledge-based explanations:* These explanations are generated by inferring some formal rules and facts in the knowledge base. For instance, a recommendation engine can offer a camera with less memory and resolution by referring to the rule that states “Less memory $\wedge$ lower resolution $\rightarrow$ cheap” [41]. Such rules need to be given to the system, and they can be derived from a decision tree modeling the system’s or user’s behavior. In other words, decision trees could be utilized to learn why the underlying decision is made from the data, and the rules extracted from the constructed decision tree can give insights on how the system works to the user as an explanation [19].
- *Example-based explanations:* Based on historical data or previous experiences, a system can generate some explanations by generalizing past behaviors/patterns for a given new situation [48]. For example, assume that a food recipe consisting of sugar-free ingredients was recommended to a diabetic person by a recommender system that recommends food to ill people, and the results were satisfactory. If a new diabetic person joins the system, it might generate the following explanation alongside its recommendation “Diabetic people are often satisfied with this food recipe with sugar-free ingredients.”.
- *Content-based explanations:* Inspired by the content-based recommendation approach, the system can analyze the features of the items appreciated by a particular user and extract the preferred values for those features to explain the recommended item to that user [41]. For instance, the system can generate an explanation such as “This food recipe contains mozzarella, so you might like it.” if the user previously liked the food recipes that contain mozzarella specifically.
- *Contextual explanations:* External factors affecting the decision could be used to generate such explanations. For instance, “Today fish is fresh. It has just arrived. Therefore, I recommend creamy salmon pasta.” [34].
- *Contrastive explanations:* A recent review by [31] provides empirical evidence supporting the practical utility of everyday contrastive explanations, “comparing a certain phenomenon with a hypothetical one” [48]. While asking about a certain choice, someone may think of alternatives and wonder why those were not recommended with respect to the given one. Contrastive explanations focus on the difference between the current choice and alternative ones. For instance, “We were going to recommend you a healthier option, which is Turkish Salad instead of American Salad that contains a substantially higher amount of fats.”.
- *Counterfactual explanations:* Like contrastive explanations, counterfactual explanations focus on the differences between alternative options. However, these explanations rely on hypothetical factors instead of factual factors [34]. For instance, “If you did not have an allergy to seafood, I would recommend you a salmon salad. However, now I have to recommend you a turkey salad.”.

The first three types in the list above, namely user-centred, knowledge-based and example-based, differ in the type of argument to convince the user. The first relates to what the user previously stated as preference or goal, whereas the second refers to external knowledge, in our case from a food expert. The third refers to an analogy with what other people in a peer group have chosen. By contrast, the fourth type, content-based explanations, is based on features derived from the recommendation itself. One can match those features with user preferences, external knowledge, or examples from peers, to make an argument, as mentioned before.

The fifth type picks contextual factors to focus the argument upon. In our case, the time of day determines the type of meal (breakfast, lunch, dinner). In that sense, most of our explanations are implicitly contextual. The final two types of explanations focus on the fact that explanation should help people make a choice among two or more alternatives. A contrastive explanation signals the differences between existing alternatives, whereas a counterfactual explanation signals the differences between the given selection criteria and other potential, but non-actual, selection criteria. In implementation, we have to make a combination of explanation generation strategies, and use those arguments that are most convincing in a given situation. For example, if a knowledge-based explanation fails to convince the user, an explanation based on examples from the same group of users, may work better. There are also interesting cultural differences. A user-based explanation may work better in an individualistic culture, for example. The proposed combination of strategies targeted to the food domain is novel, even if the component strategies (user-centered; content-based) have been used before.

## Proposed approach

**Fig. 1:**
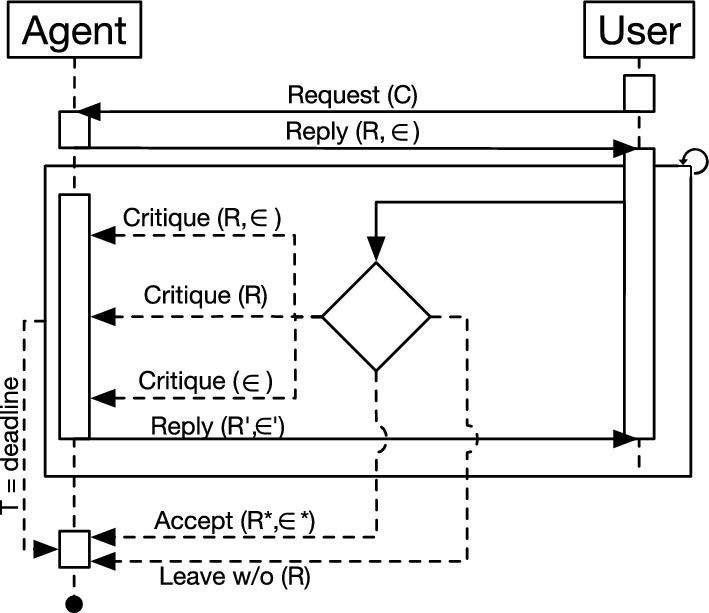
FIPA description of the negotiation protocol where C corresponds to user constrains, R is a recipe recommended by the agent and $\epsilon$ is an explanation that comes with the recipe

Our earlier study presented in [6] proposes a design of an interaction protocol for explainable NVC. In particular, it provides recommendations for recipes seeking to balance the long-term user’s diet while matching their immediate preferences. The approach presented in this study relies on the protocol presented in [6] to engage a dialogue between the user and the system. Recall that our previously developed explanation system was “static” with only nutritional factors determining the explanations. Following the feedback we acquired from previous experiments, we improved the explanation generation strategy in a more dynamic manner to enhance the dialogue between the user and agent. The protocol (see Fig. 1) is characterized by the user expressing their preferences and constraints to the NVC, which in turn replies by recommending an appropriate recipe, along with its explanation.

In the context of food recommendation, the user first reveals their constraints (*C*), which may consist of the ingredients the user may be allergic (e.g., milk, peanuts) to; the (dis)liked ingredients (e.g., specific meat/vegetables); and the desired type of cuisine (e.g., Middle Eastern, Italian, French). After receiving the user’s constraints, the agent recommends a recipe (*R*) along with its explanation ($\epsilon$). The user can *accept* *R*, *leave* without an agreement, *criticize* *R*, $\epsilon$, or both. When the user makes a critique, the agent can revise its recommendation/explanation, generating ($R'$), ($\epsilon '$), or both. This interaction continues in a turn-taking fashion until reaching a termination condition (i.e., Accept or Leave w/o Recommendation) or the time deadline is reached.

In our current implementation, a user can criticize the given recommendation by referring to pre-structured critiques as follows, where *Y* denotes one of the ingredients chosen by the user. (1) I ate *Y* recently, (2) I’m allergic to *Y*, (3) I don’t like *Y*, and (4) I want to give custom feedback. Similarly, the user can criticize the explanations communicated alongside the recommendations with the pre-defined statements such as (1) The explanation is not convincing, (2) The explanation does not fit my case, (3) The explanation is incomplete, (4) The explanation is not clear enough, and (5) I disagree with the explanation.

In the following section, we look into to the ontology database that the recommendation engine takes advantage of while calculating the recommended recipes.

### Ontology structure

**Fig. 2:**
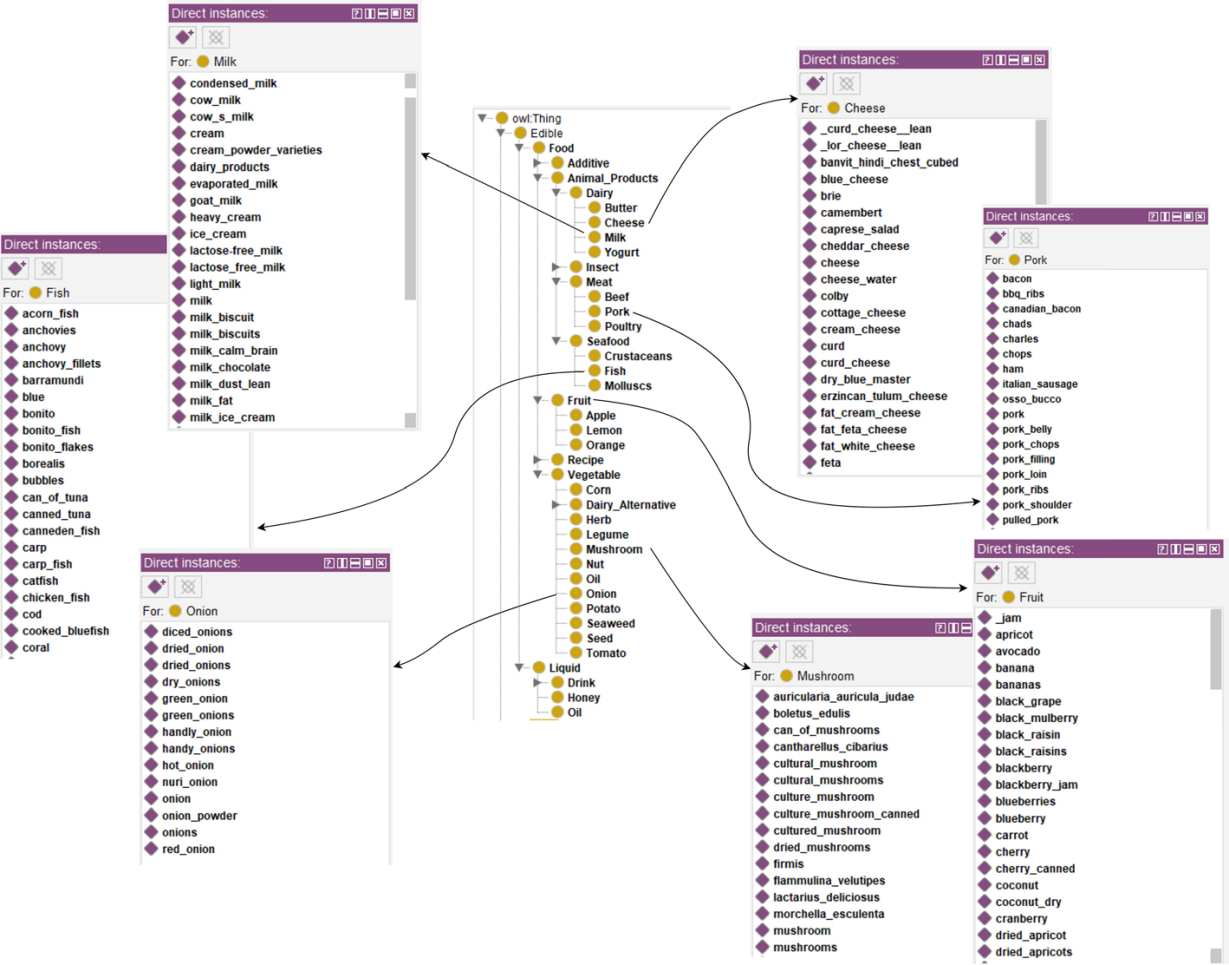
Protege view of food class

The system incorporates an OWL-based Ontology database that includes ontological concepts to represent *users* and *food ingredients*. The *User* concept characterizes the individuals and their eating habits, including any allergy, religious, and lifestyle restrictions. The food concept is characterized by recipes and ingredients that are grouped in classes (e.g., cow-hearts, cherry tomatoes, etc. are grouped under the category of *Tomatoes*). A comprehensive view from *Food* concept in the Protege is shown in Fig. 2.

**Fig. 3:**
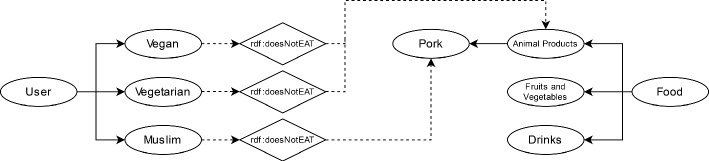
Broad overview of the ontological structure for food concept

We establish the object property of *doesNotEat* to identify which food ingredients the user would/should avoid as seen in Fig. 3. The limitations, such as the prohibition of pork for Muslims, are represented by linking object properties (depicted as diamonds) to both the “User” and “Food” concepts. The system verifies whether a particular user class would/could consume a given ingredient class by the *doesNotEat* relation between users and food ingredients. We utilized a compact and localized recipe dataset [2] to build the ontology instances by fitting the ingredients into the respective concept structure manually. We annotated the recipe ingredients by the classes of ingredients within the ontology. A final filter on recipes with incomplete information leaves 1.3K recipes to recommend.

### The baseline recommendation strategy

In this section, we explain the main recommendation strategy of the food recommender system under the following outline. Section 3.2.1 explains the initial filtering and scoring of the food recipes under various modules. Then, Sect. 3.2.2 elaborates the utility function used in determining which recipes to recommend from a healthiness perspective. Finally, 3.2.3 outlines the calculation of the user satisfaction score used in the utility estimation of the recipes.

#### Filtering and scoring recipes

To analyze the applicability of the designed protocol, we have developed a basic recommendation strategy relying on filtering and scoring the recipes concerning the user’s constraints and healthiness (see Algorithm 1). First, the NVC agent filters the recipes according to the user’s eating habits/constraints via ontology reasoning on what (classes of) ingredients the user would not consume (Lines 1–3). Assuming that the user is vegan, the NVC agent first filters the recipes containing animal-related products. Then, if the same user specifies that they do not like “zucchini”, the NVC agent removes the recipes containing zucchini from the remaining candidate list, $R_u$. In turn, the utilities of the remaining candidate are calculated by considering both healthiness and their alignment with the user preferences. Then, the recipes are sorted according to the calculated utilities (Lines 4–5).^3^ The recipe with the highest utility is taken as a candidate recipe, and the system retroactively generates an explanation in line with the recipe’s properties (Lines 6–7). This candidate recipe and its corresponding explanation are given to the user.

When the NVC agent receives feedback from the user regarding the recipe, $F_r$, it filters the candidate recipes according to the updated constraints given by the feedback and selects the highest-ranked recipe similarly (Lines 10–15). When the NVC agent receives feedback from the user regarding the explanation, $F_{\epsilon }$, it simply generates a new explanation with the underlying recipe (Lines 16–18).

**Algorithm 1:**
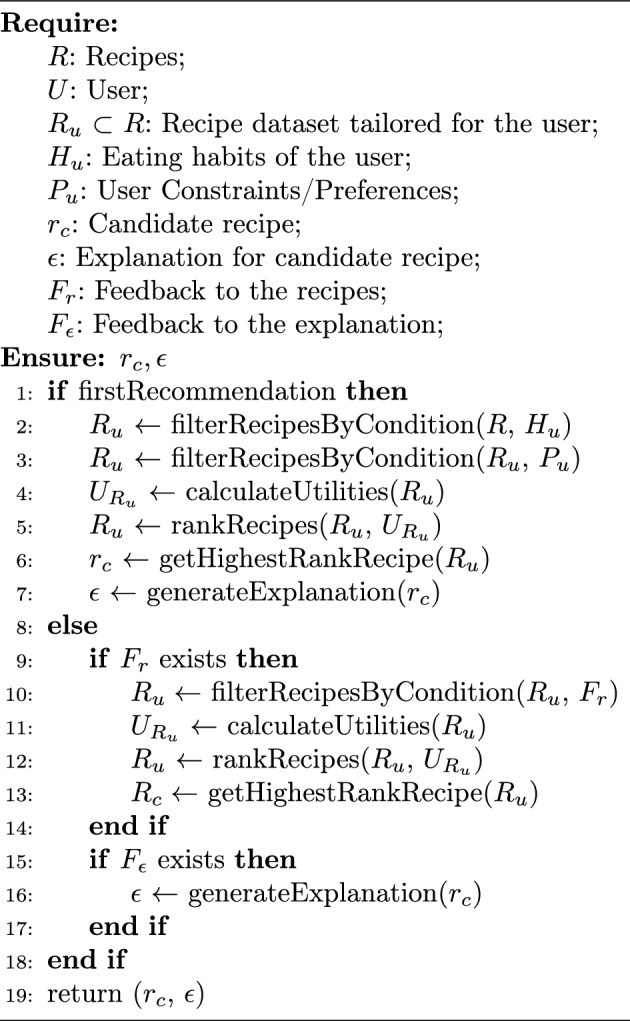
AgentDecisionFunction

#### Utility estimation

To select the suitable recipe, this study relies on multi-criteria decision-making [25]. Multi-criteria decision analysis allows decisions among multiple alternatives evaluated by several conflicting criteria [49]. The adopted multi-criteria decision analysis is done by ranking recipes through a multi-criteria function. The multi-criteria function gives each recipe a score in the dataset. One of the main advantages of using a mathematical function is the transparency of the function and its outcomes. This feature is well suited for our proposed NVC due to the explainability of the generated behavior.

The overall utility of the recipes, based on the multi-criteria, is computed by considering three criteria: Active Metabolic Rate (AMR) score, nutrition value score, and users’ Satisfaction score. The final score of the recipes is the weighted sum of the score provided by each module as presented by Eq. 1 where $w_a, w_n, w_u$ denote the weights of each AMR score, nutrition value score, and users’ satisfaction score, respectively. Note that each score is normalized to ensure that the overall score is ranged within [0,1].

1 $$\begin{aligned} recipeScore = w_n * nutrientsScore + w_a * amrScore + w_u * UsersScore \end{aligned}$$

The nutrient-based score is calculated according to the nutritional information of the recipes, such as proteins, lipids, carbohydrates, cholesterol, sodium, and saturated fats. These nutrients have respective recommended amounts for a healthy life [42]. In this work, we take into account the nutrition intake limits specified by the WHO organization.^4^ Accordingly, the nutrition-based score is calculated as seen in Eq. 2, where each nutrition score is calculated according to Eq. 3. We assume that consuming less than each nutrient’s minimum amount ($min_n$) is better than its maximum amount ($max_n$). By following this heuristic, the individual score of each nutrient is calculated.

2 $$\begin{aligned}{} & {} \begin{aligned} nutrientScore(recipe) = score(pro) + score(lip) + score(cb) +\\ score(ch) + score(sod) + score (sat) \end{aligned} \end{aligned}$$

3 $$\begin{aligned}{} & {} score(n) = {\left\{ \begin{array}{ll} 5 &{} \text {if } n \in [min_n, max_n]\\ 3 &{} \text {if } n < min_n\\ 1 &{} \text {else } \end{array}\right. } \end{aligned}$$

AMR is the number of calories a person must consume daily depending on height, sex, age, weight, and activity level. Such preliminary information is taken during the registration of the users. The value of AMR is based on the value of Basal Metabolic Rate (BMR), the number of calories required to keep a body functioning at rest, the person’s activity level, and the person’s desire to maintain or reduce his current weight. Table 1 presents the values to keep the current weight. To compute the AMR score based on the minimum and maximum amount of calories required for a given user available in literature [42], we rely on the same assumption of Eq. 3 that is consuming fewer calories than required ($score = 3$ ) is better than consuming more calories than required ($score = 1$). In addition, when the amount of calories computed is between the minimum and maximum amount of calories, the score is set to 5.

**Table 1:** Daily recommended kilocalories (kcal) intake to maintain weight [42]

Activity level	Daily calories
Too little exercise	$calories = BMR *1.2$
Light exercise	$calories = BMR * 1.375$
Moderate exercise	$calories = BMR * 1.55$
Strong exercise	$calories = BMR * 1.725$
Very strong exercise	$calories = BMR * 1.9$

Conventionally, the most used formula to compute BMR is the Harris equation [22] with Eq. 4 and 5, for men and women, respectively. The authors estimated the constants of Eq. 4 and 5 by several statistical experiments [22].

4 $$\begin{aligned} BMR= & {} 10 * weight + 6.25 * height - 5 * age + 5 \end{aligned}$$

5 $$\begin{aligned} BMR= & {} 10 * weight + 6.25 * height - 5 * age + 161 \end{aligned}$$

#### User satisfaction score

The user satisfaction score is calculated by considering the recipe’s popularity among all users and the current user’s preferences equally. For the recipe’s popularity, we use the ratings the other users gave between [1, 5]. These values are normalized to [0, 1]. Meanwhile, regarding the user’s preferences, we check how many ingredient classes are considered to be liked by the user. Here, to determine whether an ingredient is liked or not, we can use explicit feedback from the user as well as rely on user profiling to predict whether the given ingredient is likely to be preferred to be consumed. Here, we use Jaccard Similarity [5] to estimate individual user satisfaction (the rate of the preferred ingredients over the number of all the ingredients of a given recipe).

Let us assume the user-submitted his preference for some ingredients (e.g., ingredients; $i_1$, $i_2$, $i_3$) and we have a recipe such that $R = i_1, i_2, i_5, i_6$ (where $i_5$ and $i_6$ are ingredients the user has no preference for). Each ingredient that exists with the liked constraint is considered to be 1 and 0 otherwise. The mean of this operation is 0.5, which is effectively the score of *R* for this user. For all the recipes, the scores are then max-normalized to place the values between [0, 1], resulting in a relative level of importance for the given recipe. For instance, let us assume that the system knows that the user likes the ingredients $i_1$, $i_2$, and $i_3$ and calculate the score of a recipe consisting of the following ingredients:$i_1, i_2, i_5, i_6$. The individual user satisfaction would be 2/4, according to Jaccard similarity. If the overall user rating of that recipe is equal to 4 out of 5, then the overall score would be equal to 0.65 ((0.5+0.8)/2).

### Post-hoc explanation generation strategies

This study proposes a Post-Hoc explanation generation technique to improve the transparency and the sociability of the food recommender system to nudge the users to consume healthier food. Section 3.3.1 elaborates on our use of decision trees to explain given food recommendations and Sect. 3.3.2 explains the contrastive food recommendations, where we offer an alternative and explain the differences between. Finally, Sect. 3.3.3 explains how all these approaches are combined.

#### Item and user based explanations

Decision trees are often used for decision support systems because they are simple and intuitive models that can be easily understood and visualized. They can explain the reasoning behind AI predictions or decisions in a more straightforward form than an otherwise black-box model [4]. In order to discover the important features significantly influencing users’ decisions (e.g. carbohydrates, protein, etc.), a decision tree is constructed from a labelled dataset (see Line 1 in Algorithm 2). When we employ the user-based explanation generation method, the decision tree is constructed from historical data in which recipes are labelled with *all users’ decisions* (i.e., accept or reject). Conversely, the item-based explanation generation approach utilizes the decision tree constructed from a set of recipes labelled according to *the current user’s constraints and feedback*. For that tree, filtered and low-scoring recipes are negatively labelled (-1), recipes that aligned with the user’s constraints are positively labelled (+1) and the rest is labeled neutrally (0). After sorting features with respect to their importance (Line 2), we choose three of them to generate an explanation for the given recipe (Lines 3–4 in Algorithm 2).

**Algorithm 2:**
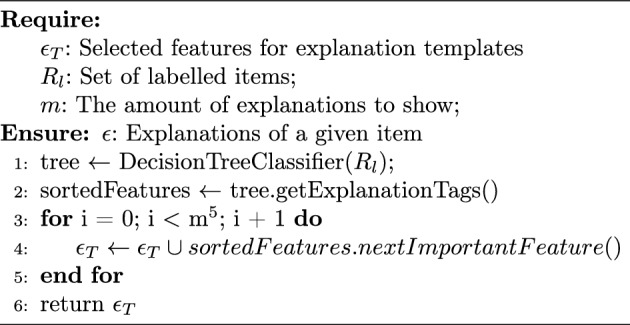
Item-Based/User-Based Explanation Generation

**Fig. 4:**
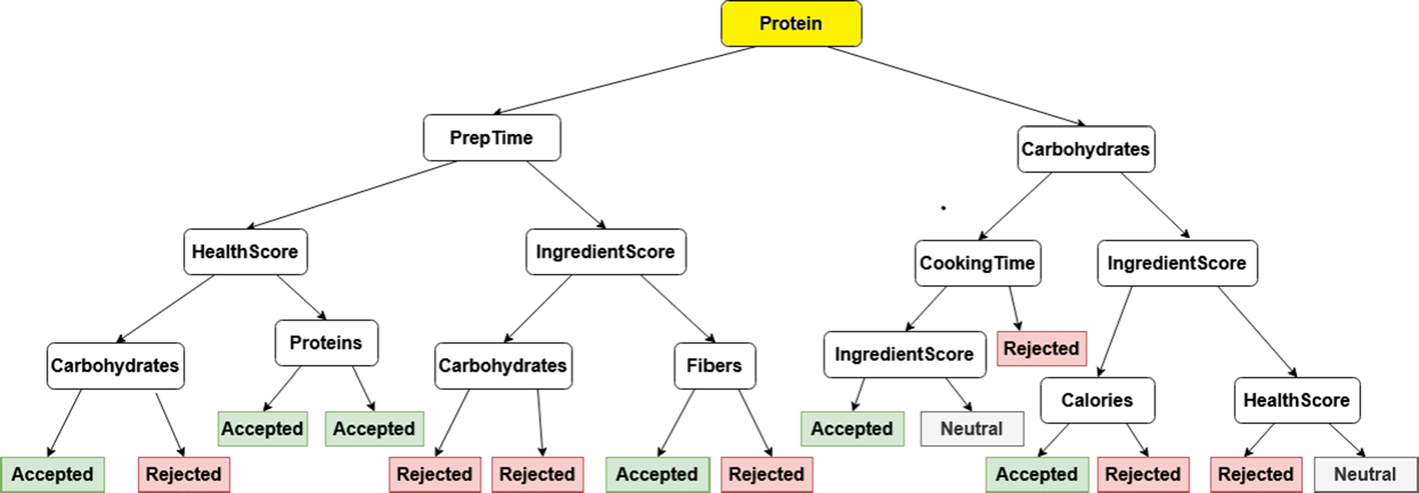
Sample tree for item-based explanations where “protein” is the most informative feature regarding the information gain

**Fig. 5:**
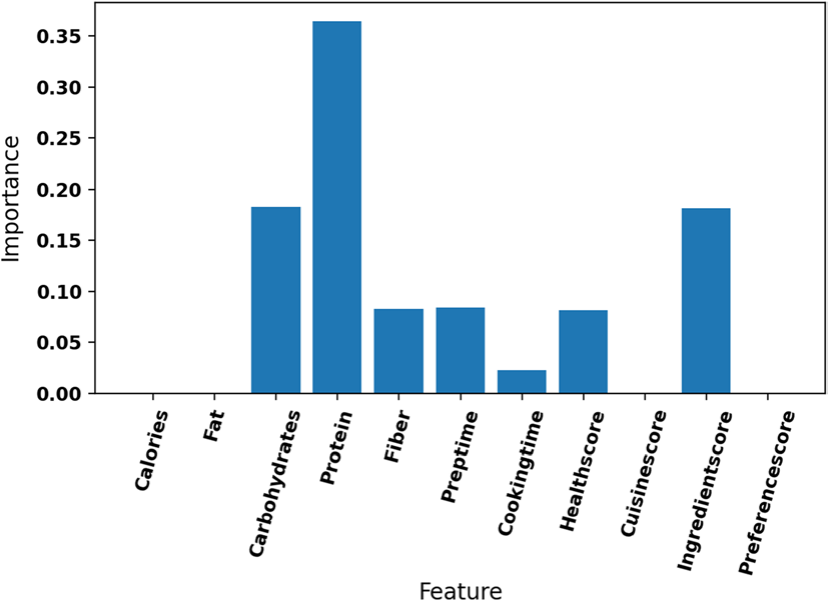
Corresponding feature importances for the Fig. 4

Figure 4 illustrates a sample item-based tree from one of the live experiment participants’ data. For this participant, one could observe that the protein is the most important decision factor for the constructed tree, as it is also visible on Fig. 5 as well.

#### Contrastive explanations

Additionally, we generate contrastive explanations as outlined in Algorithm 3. First, we select a recipe that is similar to the recommended recipe but it’s *recipeScore* is less than the recommended one. To do so, we utilize a pool of filtered (i.e., eliminated from the recommendation pool due to the user constraints/preferences) and/or low-scoring (i.e., not healthy or not tasty for the given user) recipes. We employ the Jaccard Similarity metric [26] to determine the recipe similarity based on their ingredients. From this candidate set of recipes, we choose the one whose similarity with the current recommendation is maximum (Line 1). Then, we compare features of the chosen counter recipe with those of the recommended recipe one by one. If the feature of the chosen recipe has a lower score for healthiness or user satisfaction, we added them into negative feature set, $\epsilon ^{-}$, (Lines 2–4); otherwise, inserted into positive feature set, $\epsilon ^{+}$, (Lines 5–7). Those features will be used to build a contrastive explanation sentence highlighting the positive side of the recommended recipe while sending away the contrastive recipe by emphasizing its negative sides.

**Algorithm 3:**
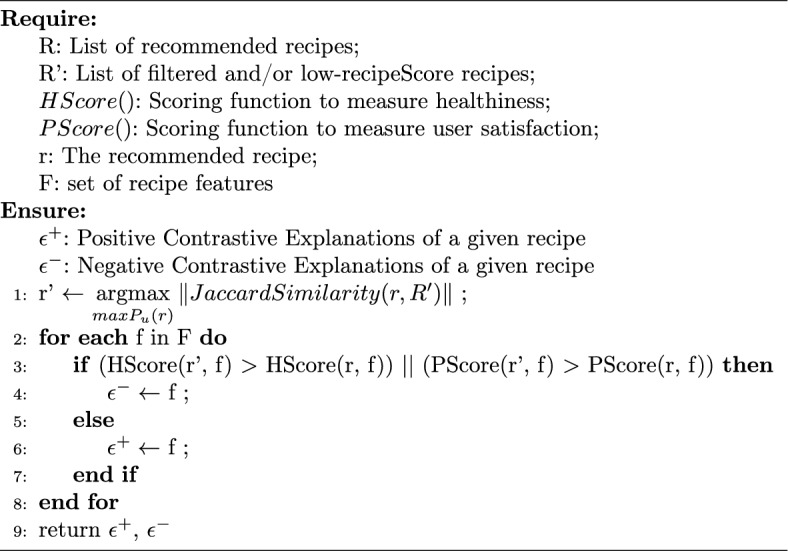
Contrastive explanation generation

#### Grammar structure and visual components

**Fig. 6:**
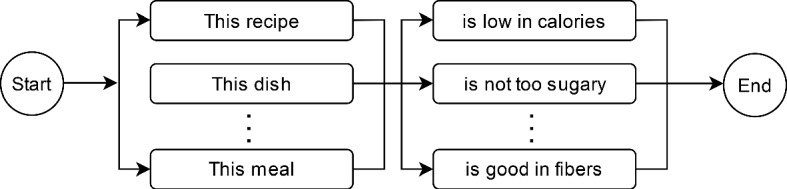
Grammar structure of the item/user based explanations

**Fig. 7:**
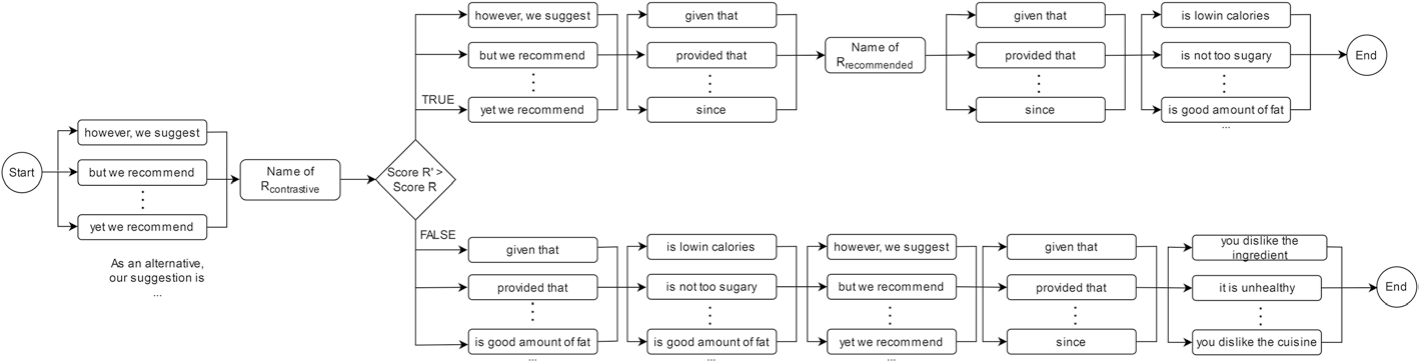
Grammar structure of the contrastive explanations

From the features acquired by the methods explained in the previous sections, we generate a sentence using the pre-defined grammar-based structure. The structures are composed of two variants: one for the user / item-based explanations is shown in Fig. 6 and the other one for contrastive explanation in Fig. 7. The phrase repository of the system consists a set of phrases for each decision factor (e.g., for protein: “...provides sufficient protein...”), and other types of phrases such as subject and noun (e.g., “...this recipe...”). The user/item-based explanations are alluring sentences about each positive feature. They are intended to be brief and pithy, whereas contrastive explanations aim to create a comparative explanation with a worse alternative (which can be longer).

**Fig. 8:**
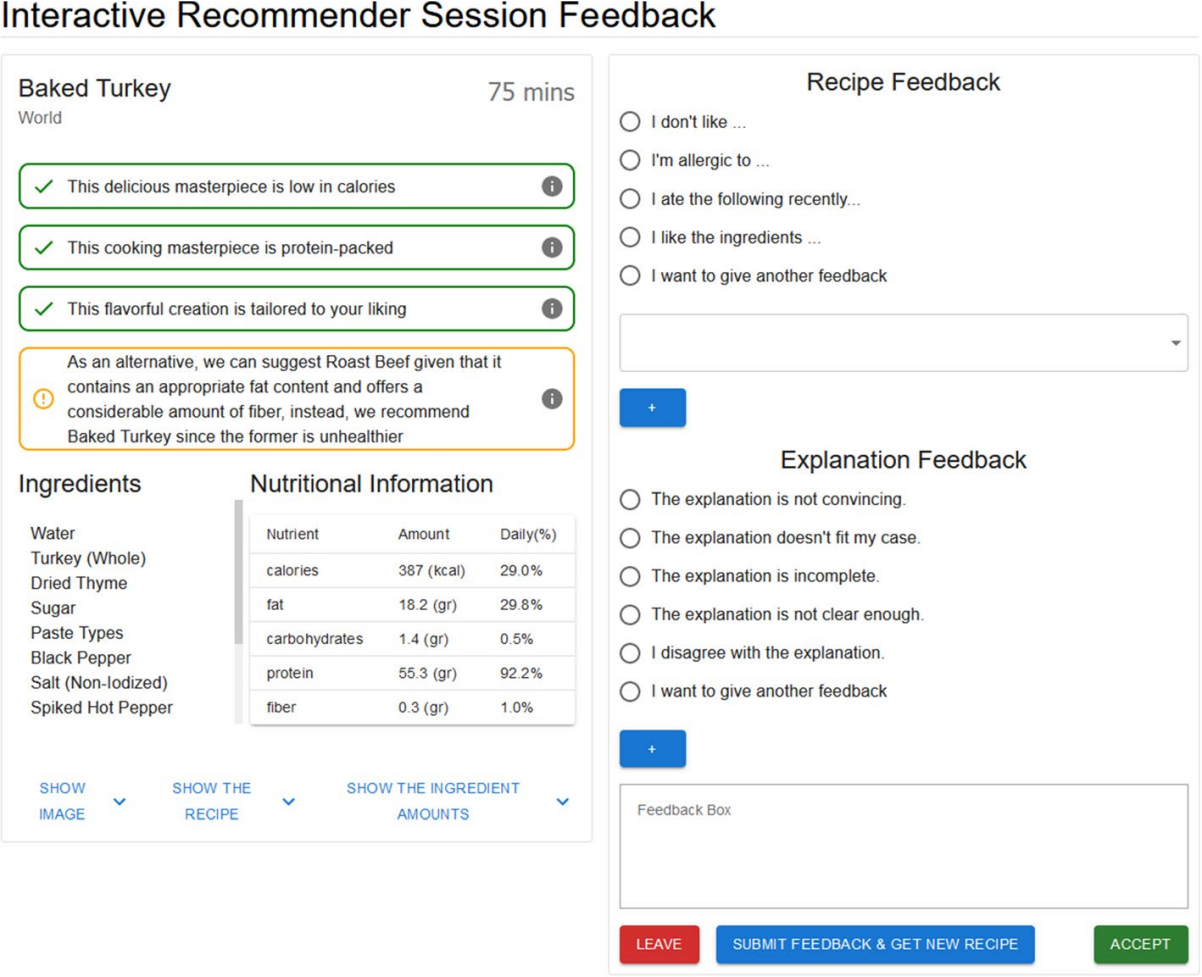
A sample of the food recommendation interface with explanations

Figure 8 shows the novel interface developed to display these explanations. We added visual aspects of explainable recommendations given the success of “graphics” in explaining recommendations [30]. The health-oriented explanations are shown in a green box. Contrastive explanations are outlined in yellow. Additionally, we present nutritional factors related to food to the user.

## Evaluation

To evaluate the performance of the proposed negotiation framework equipped with enhanced explanations, we conducted tests via a web-based interface for food recommendations. The experimental setup and participants are presented in Sects. 4.1 and 4.2, respectively. Consecutively, Sect. 4.3 reports and discusses the experimental results elaborately.

### Experimental setup

**Fig. 9:**
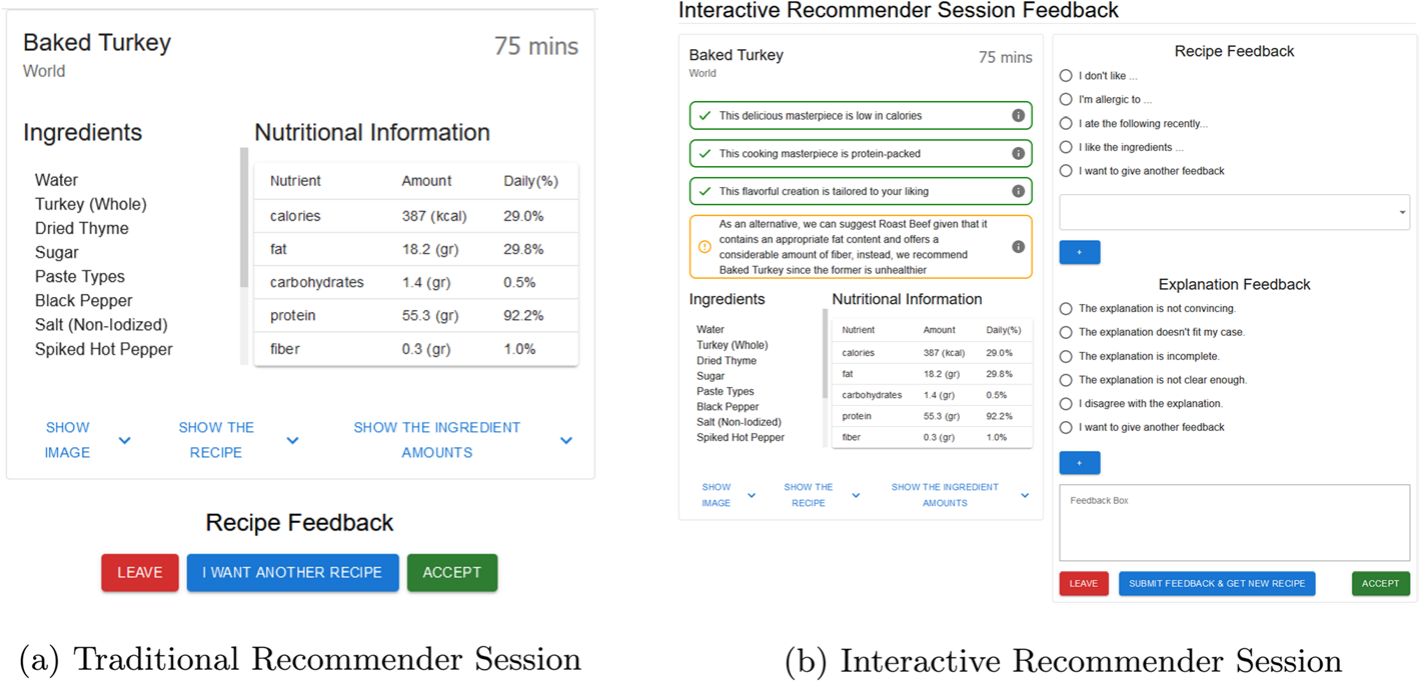
traditional and interactive recommendation sessions

To assess the acceptability and effectiveness of the proposed negotiation-based recommendation framework, we asked participants to experience two variants of food recommender systems: (i) *traditional recommender* where the the system provides solely a recommendation (picture and recipe) without any explanation, leaving the user to accept it or ask for a new recommendation, and (ii) *interactive recommender*, where the original explanation-based negotiation approach is adjusted to an interactive setting, providing explanations for the recommendations and allowing users to give feedback (i.e., approvals and critiques of the recipe and/or explanations). It is worth noticing that we revised the Web participant interfaces in both conditions based on the feedback received in the earlier study presented in [6]. We improved how the food recipes and their supportive explanations are displayed to communicate the explanations more effectively and diminish the effect of factors irrelevant to the quality of explanations, such as pictures. Nutritional information and main ingredients are shown directly alongside several types of explanations. Conversely, as visible in Fig. 9, a picture of the food as well as the details of the recipes are not directly displayed, but available only via an additional click.

We follow the following steps in our experiments^5^. Before conducting the experiments, every participant completed a pre-survey and registration form to provide information about their gender, age, height, weight, level of physical activity, dietary preferences, and any allergies they might have. This information concurs to estimate the healthiness score of recipes recommended to the participant (see Sect. 3.2). To reduce the learning effect among the sessions, the participants were split into two “groups”, inverting the starting settings order. A three-minute break was given between the two sessions. Initially, we scheduled a longer break. However, in our pilot experiment, we received negative feedback about the too-long waiting interval.

**Fig. 10:**
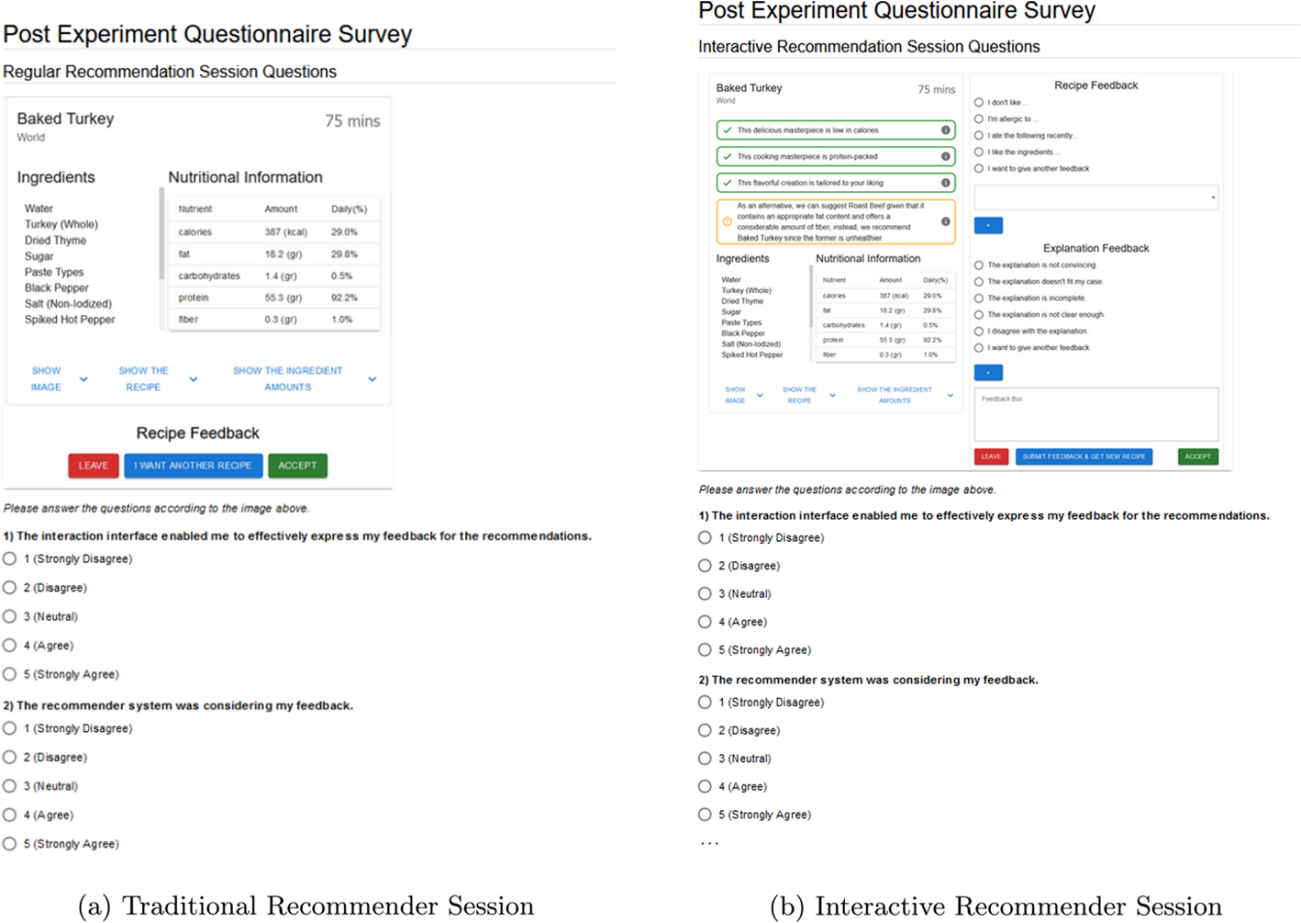
Traditional and interactive recommendation sessions questions

Following the completion of the experiment, the participants are asked to fill in a questionnaire that primarily comprises 5-point Likert scale questions to assess their experiences in both sessions (one questionnaire per session). The questionnaire follows a within-subject design [29] to gather participants’ insights regarding the system’s success. To facilitate recalling their experiences and differentiate the sessions, a picture (screen capture) of the given system’s setting is displayed at the beginning of the questionnaire page (see Fig. 10). Finally, additional 5-point Likert scale questions were asked to the participants about their perceptions of the received explanations during the Interactive system.

### Participants

**Fig. 11:**
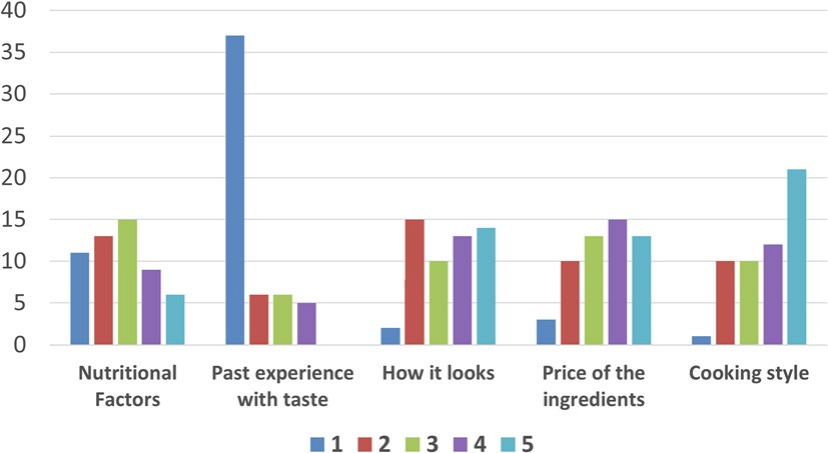
Histogram analysis of the pre-survey questionnaire

In total, there were 54 participants (19 female, 35 male) with diverse backgrounds and age groups took part in the test. The mean age of the attendees is 26.31 years old (with a minimum of 19 and a max of 58 years old). The participants were requested to order the importance of five criteria, relative to a given food recommendation: “Nutritional factors”, “Past experience with taste”, “How it looks”, “Price of the ingredients”, and “Cooking style”. Figure 11 shows the histogram analysis of the questionnaire. The participants ranked these factors on a scale of 1 to 5, with 1 being the most important factor. One could observe that the majority of the participants (i.e., 69 % of the participants) ranked past experience with the taste of such food to be the most crucial factor in deciding their food recipes to cook, whereas 21% of the participants marked nutritional factors to be the most important. Conversely, 39% of the participants marked cooking style as the least important factor, whereas the food’s appearance was rated the least important by 26% of the participants.

### Experimental results

The success of the self-explanatory systems is usually measured under two categories of metrics; subjective and objective metrics [23, 24, 48]. Objective metrics are metrics derived from the participant actions within the experimental setup, such as success rate (i.e., percentage of sessions ending with an agreement), number of rounds per session, healthiness level of the accepted food recipe, and annotator analysis of possible misunderstandings and feedback given during the Interactive session. Subjective metrics denote the participant scores for the post-experiment questionnaire (see Fig. 15 below). The subjective evaluation questions are about perceived effectiveness, level of detail, user satisfaction, understandability, informativeness, and convenience, meaning that the explanations are appropriate relative to the stated user preferences and constraints. In addition, we asked about the general idea of receiving explanations in addition to recommendations.

We first analyzed the number of sessions that ended successfully. Out of 54 sessions, only two traditional and one Interactive session ended without any agreements. It is worth noting that the participant who failed to find agreement with the Interactive system also couldn’t find one with the traditional system.

**Fig. 12:**
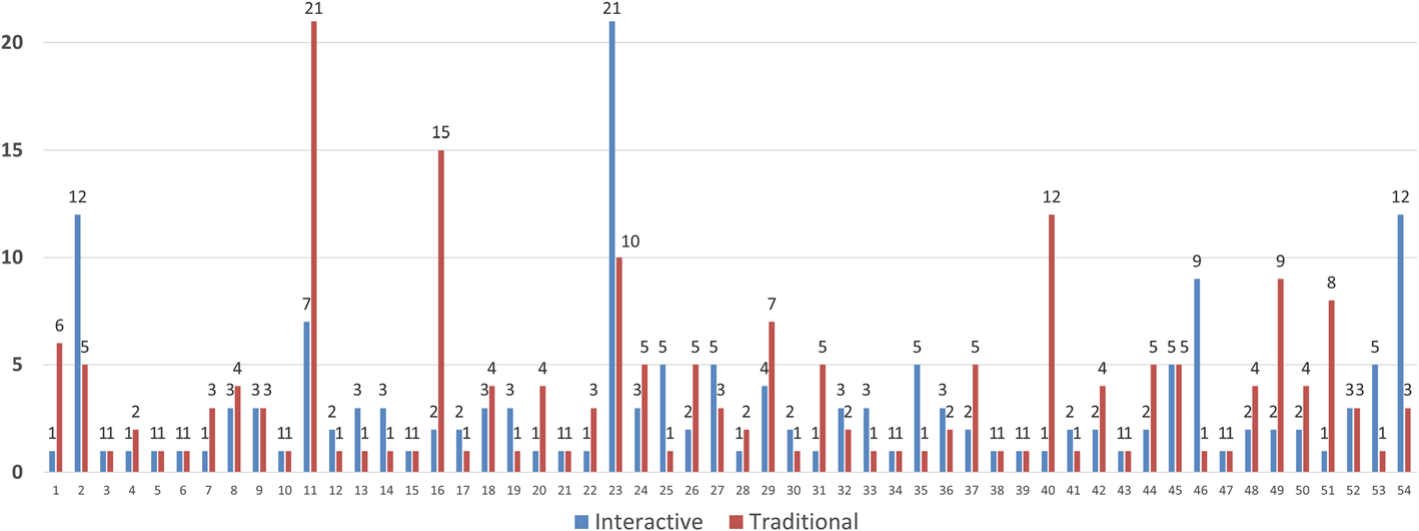
Total number of rounds per participant, for each interaction type

Moreover, participants reached an agreement in the third round on average when they engaged in the Interactive session (i.e., average=3.1 standard deviation= 3.5). In contrast, they accepted the given offer in the forth round on average for the traditional session (i.e., average=3.6 standard deviation= 3.9). Total number of rounds per each participant in each session can be seen in Fig. 12 where the red and blue bars denote the total number of rounds for the traditional and Interactive sessions, respectively. Compared to traditional recommender session, 18 participants had more interaction in the Interactive session, whereas 22 participants required more rounds to find an agreement in the traditional sessions. Another 14 participants finished the interaction in the same number of rounds. The results are not normally distributed according to Kolmogorov–Smirnov test of Normality ($p=<0.001$), therefore we applied the corresponding non-parametric Wilcoxon Signed-Rank Test ($p=0.347$). Ultimately, we can infer that the interactive recommender systems do not necessarily take more rounds to reach an agreement, as might be expected.

**Fig. 13:**
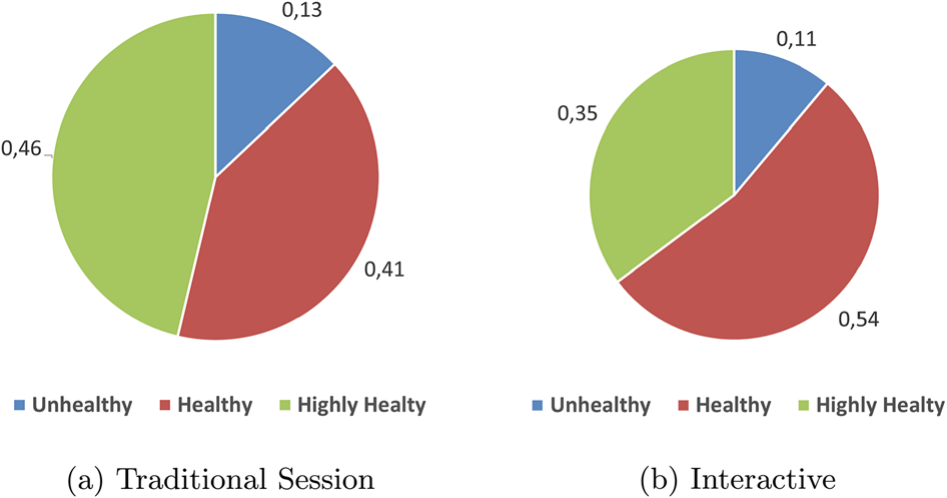
Percentage of healthiness level of the agreement

For the Interactive session, 19 participants accepted recipes of—what we classified according to Eq. 3, the recipes that are labeled “5” as—highly healthy foods; 29 participants preferred healthy foods, and six accepted unhealthy food recipes. For the traditional session, on the other hand, the participants accepted 25 highly healthy options and 22 healthy options; in contrast, seven participants went for unhealthy options. These results are illustrated in Fig. 13. That shows that the Interactive and traditional sessions are similarly effective in meeting the objective of recommending healthy foods. When the Chi-square statistical test was applied, we observed that there was no statistically significant difference between the distributions ($p=0.40$). Recall that the recommendation strategy itself is the same in both sessions.

The aforementioned results concerning the total number of rounds per session indicate that 18 participants ended the session in the traditional session earlier than the Interactive one. It is possible that they enjoyed exploring the system more in the Interactive system. Since the recommendation strategy employs a time-based concession strategy, the longer it endures, it may offer less healthy food relative to its previous offers. As a result, traditional sessions may end up with healthier food recipes compared to the Interactive system in some cases. On the other hand, there are less unhealthy food recipes agreed by the participants in the Interactive session.

Out of 54 participants, the system received the following evaluative feedback for Interactive sessions:

- “The explanation doesn’t fit my case”, from 4 participants,
- “The explanation is not convincing”, from 4 participants,
- “The explanation is not clear enough”, from 2 participants,
- “The explanation is incomplete”, from 1 participants,
- “I disagree with the explanation”, from 1 participants.

Additionally to our given feedback options, which were all negative, participants utilized the custom feedback option to compliment the explanations: “The explanation is acceptable” or “The explanations are enough for me”.

Furthermore, we analyzed the users’ responses to the post-test survey to examine how they perceived the traditional and Interactive recommendation system. Since each participant experienced both sessions and the questions are the same for both, we performed a within-analysis statistical comparison test. The data is not normally distributed which is one of the main assumptions made by the pairwise T-test. Thus, we apply the corresponding non-parametric test called the Wilcoxon sign rank test [29]. For all tests, the Confidence Interval (CI) is set to 0.95, $\alpha = 1-CI = 0.05$.

**Fig. 14:**
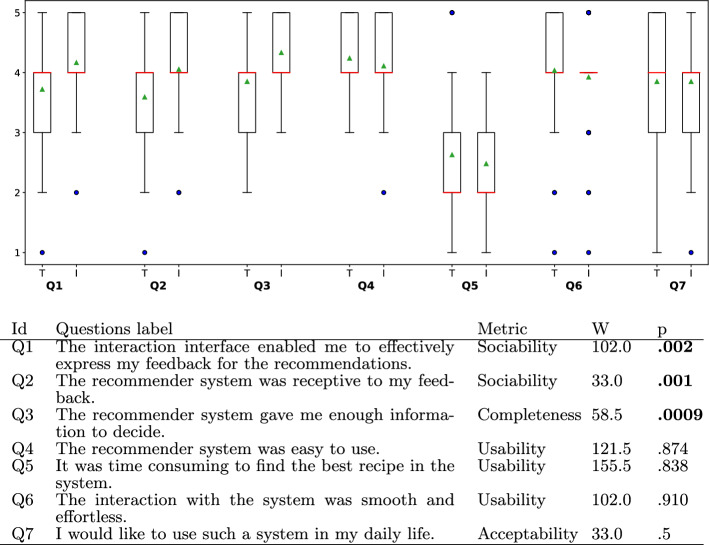
Box plot and *p*-values of comparative analysis of subjective questions between traditional and interactive sessions. Significant results are shown in bold

Figure 14 shows the box plot of the comparative questionnaire between the traditional (*R*) and the Interactive (*I*) session, respectively. The orange lines represent the median, the triangles in green the means, and the small blue circles the outliers.

The analysis in the box plot shows that there is a significant improvement for the Interactive sessions, especially for questions $Q1(p=0.002$) and $Q2(p =0.001$). These two questions measure the system’s sociability where the feedback corresponds to the binary of choice of accept and reject for the Traditional system, and the additional live-feedback options for the Interactive system. That is, the results show that the Interactive session is statistically significantly better than the traditional session in terms of sociability. This improvement is reasonable given the additional dialogue options, such as feedback mechanisms, in the Interactive session. Q3 measures the amount of information the participants perceived to be fruitful. The added explanations were recognized by the participants to be effective, hence, here too a significant improvement has been reported ($p=0.0009$). In other words, the participants perceived that the Interactive session provided better information than the traditional session to make an informed decision.

Moreover, questions Q4 ($p=0.874$), Q5 ($p=0.838$), and Q6 ($p=0.910$) qualify the usability of the system. These values show that there is no significant difference. That means that adding an interactive dimension to the system, can still be effective and efficient. This is in line with what we found earlier about the similar number of turns. Lastly, we measured the acceptability of the two versions of the system. According to the statistical test, there is no significant difference between traditional and Interactive systems for Q7 ($p=0.5$). The average acceptability score for the Interactive session is approximately 3.85, where 3 is neutral and 4 denotes “agree”. Furthermore, we asked all participants which systems they prefer. Only a minor part of the participants (3 out of 54 participants or 6% of them) prefer the traditional one over the Interactive system. In other words, the majority favors the Interactive system (45 participants). The rest is indifferent.

**Fig. 15:**
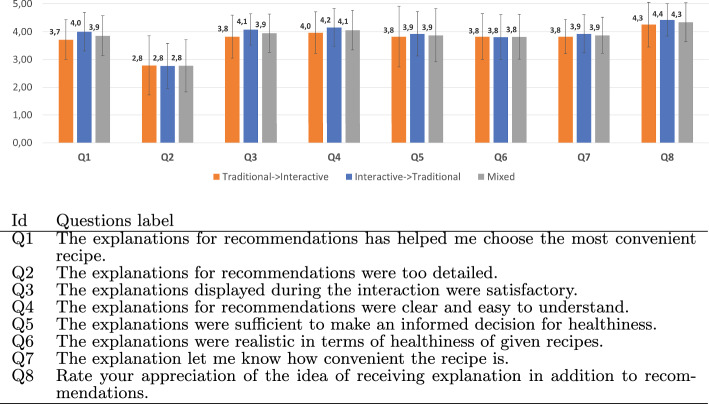
Evaluation questionnaire results, shown per order of the sessions

Apart from the comparative analysis, we also ask questions to assess the perceived quality of the explanations in our system. Hoffman et al. provide a list of so called goodness criteria for explanations [23]. Inspired by those statements, we created corresponding statements for the food recommendation system and asked each participant to what extent they agreed. Figure 15 shows the questions and the respective average scores. To examine whether a learning effect may have influenced the results, we report the average scores with respect to (1) participants who started with the traditional sessions (i.e., traditional $\rightarrow$ Interactive), (2) participants who started with the Interactive session (i.e., Interactive $\rightarrow$ traditional), and (3) all participants irrespective of the order of sessions (i.e., Mixed). It is clearly seen that the counter-balancing technique works. The results for both orderings are similar. In general, participants are satisfied with the given explanations and appreciated the idea of receiving explanations in addition to the given recommendations. They do not agree that the explanations were too detailed. In addition, they found the explanations help them choose the most convenient recipe.

**Fig. 16:**
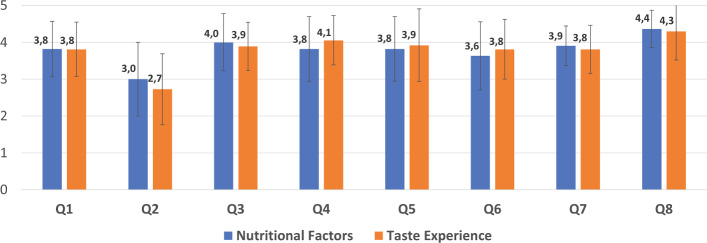
Questionnaire results per pre-survey answers

Lastly, we categorized participants based on their responses to the pre-survey question—the importance of the factors on their decision making (See Fig. 11. Since there are a few participants who found the most important factor as how the food looks, price of the ingredients, and cooking style, we only categorized the participants who voted the most important factor in choosing a recipe to be the past experience with taste and the nutritional factors of a given food. This categorization is also in line with our objectives. Figure 16 shows the score of the aforementioned explanation related questions and responses of the participants in each category. Note that since order of session does not influence the results, we only show the average scores for all participants who fit in the given category. We could not find any significant differences in their responses.

## Conclusions

The recent widespread use of opaque AI-based systems is raising questions about trustworthiness and transparency. Skepticism skyrockets when the decisions to be taken are safety-critical (i.e., AI outcomes can significantly influence people’s life and health—like nutrition). This study presents an interactive explainable recommendation framework where the system seamlessly negotiates with its users by making offers and explaining why this offer is good for them. The user can criticize the given recommendation and/or associated explanation. The proposed framework aims to improve the system’s transparency via interactive explanations. User experiments have been conducted to evaluate the proposed interactive recommender system. Participants have been asked to experience the interactive recommender and the regular one (a version of the system without explanation and feedback mechanism), as well as to fill pre- and post-experiment surveys. Although both the recommender might have recommended the same food item (in the same conditions), experimental results showed that the participants were more satisfied (in general) with the idea of explanations and appreciated generated explanations. Moreover, they perceived that the information and process for choosing their food recipe were more informative and complete in the proposed interactive recommender and felt more sociable and reactive to their feedback. Furthermore, interactive sessions performed slightly better in terms of effectiveness regarding the number of agreements and rounds.

We have tried to set-up the user studies in such a way that they give reliable results. However, our results may still suffer from limitations in the research set-up.

First, although the food recipes are derived from a real food recipe repository prepared by some nutritionists, it is worth noticing that participants were involved in a system test rather than receiving accurate food advice. We mainly compare interactive explainable recommenders with regular recommenders by keeping their recommendation strategy the same.

Second, in this research the main difference between a regular recommender and an interactive recommender system is the presence or absence of both explanation and feedback. Therefore, it is not possible to distinguish which effect, added explanation or added feedback, is responsible for the results. This signals a clear limitation in the set-up of the user experiments. In defense, consider the alternative. To separate these effects would require building a recommender system that allows negative feedback, without providing a response to that feedback in the form of a better explanation. Although theoretically interesting, that would not be a practically useful system.

Third, there is a lot of room to improve the recommendation algorithm itself. For example, we envision learning user preferences over time, and adapting the system behavior accordingly. Yet, our results already show that the proposed approach is promising.

In future work, we plan to study the effect of the precise moment in which the explanations are displayed, during the interaction and decision-making process. Recall that the current system generates explanations whenever it provides a recommendation. An interesting alternative would be to investigate so-called on-demand explanations, which are only provided when the need occurs. The need for an explanation may be signalled by a question like ‘why’ or ‘how’?.

Furthermore, we plan to measure the effectiveness of each type of explanation strategy (user-centred, contrastive, counterfactual, etc) individually, rather than as the combined whole, we have now.

The ultimate goal of our research is to refine the current recommender engine, and integrate it into an existing chatbot framework for persuading and helping a user to change eating habits over a longer period of time. The existing chatbot system is called EREBOTS [7]. The combination of long-term persuasion and coaching from EREBOTS and explainable recommendation sessions from this system, will realize a fully agentified NVC system.

## Data Availability

The data that supports the findings of this study are available upon request. However, due to privacy and confidentiality concerns, certain restrictions may apply to the availability of specific datasets. Requests for access to the data can be directed to the corresponding author. They will be subject to a data-sharing agreement to ensure compliance with relevant regulations and ethical considerations.

## Footnotes

1 https://www.who.int/news-room/fact-sheets/detail/noncommunicable-diseases.

2 https://www.cdc.gov/chronicdisease/resources/publications/factsheets/nutrition.html.

3 The details of the utility calculation are explained below.

4 https://www.who.int/news-room/fact-sheets/detail/healthy-diet, http://www.mydailyintake.net/daily-intake-levels/

5 We selected three explanations for this study. Since it's commonly considered the maximum number of items to show to a user without overwhelming them with too much information [37]
